# Effects of Pyrene on Human Liver HepG2 Cells: Cytotoxicity, Oxidative Stress, and Transcriptomic Changes in Xenobiotic Metabolizing Enzymes and Inflammatory Markers with Protection Trial Using Lycopene

**DOI:** 10.1155/2019/7604851

**Published:** 2019-10-09

**Authors:** Jin-Kui Ma, Walaa Fathy Saad Eldin, Waleed Rizk El-Ghareeb, Abdelazim Elsayed Elhelaly, Mariam H. E. Khedr, Xiang Li, Xiao-Chen Huang

**Affiliations:** ^1^School of Food & Pharmaceutical Engineering, Zhaoqing University, Zhaoqing 526061, China; ^2^Educational Veterinary Hospital, Faculty of Veterinary Medicine, Zagazig University, Zagazig 44519, Egypt; ^3^Department of Veterinary Public Health and Animal Husbandry, College of Veterinary Medicine, King Faisal University, Al Hofuf, Saudi Arabia; ^4^Department of Food Hygiene and Control, Faculty of Veterinary Medicine, Suez Canal University, Ismailia 41522, Egypt; ^5^Center for Emerging Infectious Diseases, School of Medicine, Gifu University, Gifu 501-1193, Japan; ^6^Department of Veterinary Hygiene, Faculty of Veterinary Medicine, Zagazig University, Zagazig 44519, Egypt; ^7^College of Environmental and Chemical Engineering, Zhaoqing University, Zhaoqing 526061, China

## Abstract

Pyrene is one of the major polycyclic aromatic hydrocarbons formed during heat treatment of meat and in car exhausts; however, few studies have investigated pyrene-induced adverse effects on human cell lines. This study aimed at the investigation of pyrene-induced cytotoxicity and oxidative damage in human liver HepG2 cells at environmentally relevant concentrations. Pyrene-induced changes in mRNA expression of xenobiotic metabolizing enzymes (XMEs), xenobiotic transporters, antioxidant enzymes, and inflammatory markers were investigated using real-time PCR. As a protection trial, the ameliorative effects of lycopene, a carotenoid abundantly found in tomato, were investigated. The possible mechanisms behind such effects were examined via studying the co exposure effects of pyrene and lycopene on regulatory elements including the aryl hydrocarbon receptor (Air) and elytroid 2-related factor 2 (RF). The achieved results indicated that pyrene caused significant cytotoxicity at 50 n, with a clear production of reactive oxygen species (ROS) in a dose-dependent manner. Pyrene upregulated mRNA expression of phase I enzymes including CYP1A1, 1A2, and CYP1B1 and inflammatory markers including TNF*α* and Cox2. However, pyrene significantly downregulated phase II enzymes, xenobiotic transporters, and antioxidant enzymes. Interestingly, lycopene significantly reduced pyrene-induced cytotoxicity and ROS production. Moreover, lycopene upregulated detoxification and antioxidant enzymes, probably via its regulatory effects on Air- and RF-dependent pathways.

## 1. Introduction

Polycyclic aromatic hydrocarbons (PAHs) are a group of fused-ring aromatic compounds that are released into the environment due to incomplete combustion of organic matter and are found in car exhausts, tobacco smoke, and heated meat [1]. PAHs differ in their physicochemical properties and largely vary in their toxicity, mode of action, and interactions with biological systems [2]. Pyrene is a PAH that consists of 4 fused benzene rings [3]. It is often detected in environmental and food samples at high concentrations and is regarded as one of the best candidates to study the toxicity of PAHs [4]. Pyrene is one of the major 16 PAHs according to the European Food Safety Association [5]. Once humans are exposed to such toxicants, they undergo several metabolic interactions via the xenobiotic metabolizing enzyme (XME) system leading to disruption of xenobiotic metabolic pathways [6]. The major metabolic pathway for PAHs is mainly via an aryl hydrocarbon receptor- (Air) regulated gene battery. Air regulates phase I enzymes such as cytochrome P450 (CYP) 1A1 and 1A2, phase II enzymes such as uridine diphosphate glucuronosyltransferase (UGT) 1A6, and NAD(P) quinone oxidoreductase 1 (NQO1) [7]. Several reports investigated the toxic and mutagenic effects of benzo[a]pyrene as a major promutagenic and procarcinogenic PAH in the human liver and colon cell lines [1, 8]. However, pyrene-induced adverse effects including cytotoxicity and oxidative stress and the mechanisms behind such effects are scarcely investigated.

Lycopene is one of the hydrocarbon carotenoids which is found abundantly in tomatoes, watermelon, and red carrots [9]. It is also the most abundant carotenoid that can be detected in human plasma comprising about 50% of the total carotenoid content in the human body [10]. Lycopene has potential higher antioxidant effects compared with other carotenoids such as *β*-carotene [11]. Dietary supplementation of lycopene is linked to the reduction of mutagenesis and cancer risk in several reports [12, 13]. However, the mechanisms behind the beneficial effects of lycopene against the mutagenesis and onset of cancer are still unclear. Furthermore, the protective roles of lycopene against pyrene-induced cytotoxicity and oxidative stress and the mechanisms behind such effects have received little attention.

In sight of the previous facts, pyrene was used as a model for PAH exposure and its induced adverse effects including cytotoxicity and oxidative damage were investigated using HepG2 cells as a model. Furthermore, the changes in mRNA expression of XMEs including phase I and II enzymes and xenobiotic transporters were investigated. In addition, pyrene-induced changes in antioxidant enzymes and inflammatory markers were studied. As a protection trial, the ameliorative effects of lycopene against pyrene-induced adverse effects were examined. The mechanisms behind such effects were investigated via examination of coexposure of pyrene and lycopene on regulatory elements including Air and elytroid 2-related factor 2 (RF).

## 2. Materials and Methods

### 2.1. Chemicals

Chloroform and isopropanol were of LC/MS grade and were purchased from Wako Pure Chemical (Osaka, Japan). TRI reagent, Dulbecco's modified Eagle's medium (DMEM), fetal bovine serum (FBS), pyrene, lycopene, and 2′,7′-dichlorofluorescein diacetate (DCF-DA) were purchased from Sigma Chemical Co. (St. Louis, MO, USA). Other chemicals and reagents of analytical grade were purchased from Kanto Chemical Industry (Tokyo, Japan) unless specified.

### 2.2. Cell Culture Conditions and Treatment

Human liver hepatoma (HepG2) cell lines from Cell Biolabs, Inc. (distributed by Funakoshi Co. Ltd., Tokyo, Japan) were cultured on DMEM, supplemented with 10% FBS and 1% penicillin-streptomycin mixture, in a humidified incubator with 5% CO_2_ at 37°C. When confluency was reached, the cells were exposed to pyrene at environmentally relevant concentrations (1, 5, and 50) n for 24 hr. In protection experiments, the cells were coexposed to pyrene at 50 nM and lycopene at 3 concentrations (0.1, 1, and 10 *μ*M) for 24 h. It is worth mentioning that in the preliminary experiments, the cells were exposed to pyrene for 0–48 h. However, there was no significant difference in the pyrene-induced cytotoxicity at both 24 and 48 h; therefore, the protection trials were conducted for 24 h exposure, and results of pyrene exposure for 24 h were shown in the present study. The used incubation time and concentrations for treatments were in accordance with previous reports [1, 14]. Both pyrene and lycopene were dissolved in DMSO, and the final concentration of DMSO in the medium was 0.01%, which did not show any cytotoxicity to HepG2 cells.

### 2.3. Cell Viability Assay

A CCK-8 assay kit (Dojindo Molecular Technologies, Rockville, USA) was used to determine HepG2 cell viability according to the manufacturer's instructions (*n* = 6 per treatment).

### 2.4. Reactive Oxygen Species (ROS) Measurement

HepG2 cells treated with pyrene and lycopene were stained with the fluorogenic probe DCF-DA for measurement of ROS production. The fluorogenic probe DCF-DA is used to measure the generalized oxidative stress in the cell produced by many types of reactive oxygen and nitrogen species such as H_2_O_2_, hydroxyl radicals, and peroxynitrite anions. The fluorescence intensity was measured at excitation and emission wavelengths of 485 and 535 nm, respectively, using a Wallac 1420 ARVO Mx plate reader, PerkinElmer, Tokyo, Japan (*n* = 6 per treatment).

### 2.5. RNA Isolation and cDNA Synthesis

Total RNA was isolated from HepG2 cells according to a method established before [15]. In brief, cells were lysed using the TRI reagent followed by separation of the RNA upper layer using chloroform combined with centrifugation (15000 g for 20 min at 4°C). Isopropanol was added to the clear RNA layer followed by centrifugation for precipitation of RNA pellets. The pellets were then washed with 70% ethanol and dissolved using RNase-free H_2_O. RNA concentrations and qualities were determined using a Nanodrop ND-1000 spectrophotometer (DYMO, Stamford, Conn., USA). For cDNA synthesis, a ReverTraAce® qPCR RT Master Mix with gDNA remover (Toyobo Co. Ltd., Osaka, Japan) was used as described in the manufacturer's instructions. cDNA samples were stored at −20°C for further analysis.

### 2.6. Quantitative RT-PCR (qPCR)

Gene expression of phase I enzymes including CYP1A1, 1A2, and 1B1; phase II enzymes including UGT1A6, NQO1, and glutathione-S-transferase (GST) A1; xenobiotic transporters including multidrug resistance protein 1 (MDR1) and multidrug resistance-associated protein 2 (MRP2); antioxidant enzymes including heme oxygenase (HO) 1, superoxide dismutase (SOD) 1, GSTO1, and catalase (CAT); inflammatory markers including cyclooxygenase-2 (COX2) and tumor necrosis factor-*α* (TNF*α*); and regulatory elements including Air and elytroid 2-related factor 2 (RF) were determined using real-time reverse transcriptase-PCR (qPCR). The reactions were conducted in a Step One Plus Real-Time PCR system (Applied Biosystems, Foster, CA). The PCR mixture contained 2 *μ*L of cDNA (600 ng), 5 *μ*L of Fast SYBR® Master Mix, and 5 *μ*M of each primer, with RNase-free water added to a final volume of 10 *μ*L. The reaction cycle comprised a holding stage for 20 s at 95°C, followed by 40 denaturation cycles for 3 s at 95°C and 30 s at 60°C, and 15 s extension at 95°C. Single amplicon amplification was confirmed using melting curve analysis. The absence of primer dimers and genomic DNA amplification were confirmed by agarose gel electrophoresis. GAPDH was used for normalization by the comparative ^ΔΔ^Ct method. Each experiment was represented by 6 plates/treatments. Primer sets for the selected targets were designed based on previous work [8] and are presented in Table 1.

### 2.7. Air Luciferase Assay

A luciferase assay was performed using H4IIE-XRE cells according to the method described previously [16]. In short, cells were seeded in 96-well plates in DMEM supplemented with 10% FBS. In the next day, the cells were exposed to treatments (either pyrene alone or combined with lycopene) or Sudan III (10 *μ*M) (a positive control for the activation of Air [7, 16] for 12 h). Then, the medium was aspirated off and luciferase assay was performed using a Dual Glo luciferase assay system (Promega, Madison, U.S.A.) according to the manufacturer's protocol. The activity was measured using a Wallac 1420 ARVO Mx plate reader.

### 2.8. RF Reporter Gene Assay

An RF reporter gene assay was conducted based on the protocol described before [17]. In brief, HepG2 cells were seeded into 96-well plates for 24 h with DMEM supplemented with 10% FBS. We transfected pGL4.37 [luc2p/ARE/hygro] and pGL 4.75 [hRluc/CMV] (an internal control for transfection efficiency) vectors at a 20 : 1 mass ratio using lipofectamine 3000 (Life Technologies, Tokyo) according to the manufacturer's protocol. After transfection, the transfection reagent/DNA mixture was removed and the samples solubilized in DMEM without FBS were separately applied to the transfected cells at various concentrations. Luciferase activity was assayed using a dual luciferase system (Promega, Madison, USA) according to the manufacturer's protocol. The activity was measured using a Wallac 1420 ARVO Mx plate reader.

### 2.9. Statistical Analysis

Statistical significance was evaluated using one-way analysis of variance (ANOVA) with a Tukey–Kramer honest HSD post hoc test (JMP program, SAS Institute, Cary, NC, USA) with *P* < 0.05 considered significant.

## 3. Results and Discussion

### 3.1. Biological Responses of HepG2 Cells to Pyrene Exposure

**Figure 1:**
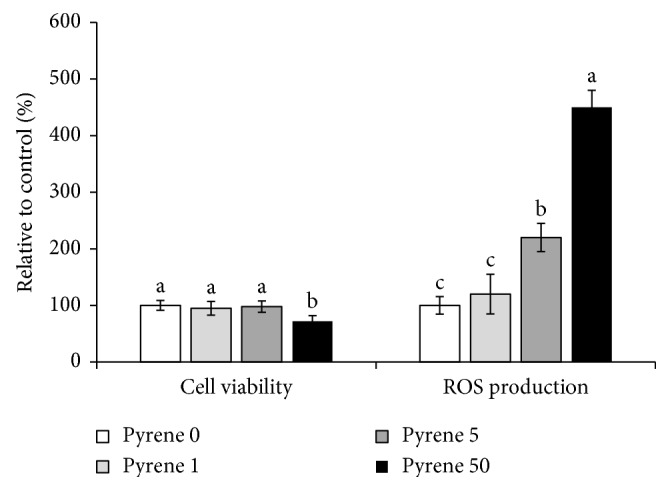
Pyrene-induced cytotoxicity and oxidative stress in human HepG2 cells. Data represent mean ± SD of pyrene-induced cytotoxicity (%) relative to the control using a CCK8 assay and pyrene-produced ROS (%) relative to the control using DCFDI as a substrate, *n* = 6. Columns with different superscript letters are significantly different at *P* < 0.05.

The achieved results indicated that pyrene had significant cytotoxic effects on HepG2 cells at 50 n causing 28% reduction in cell viability. Unlikely, benzo[a]pyrene, a promutagenic PAH, did not alter HepG2 cell viability [8, 15]. Pyrene caused a clear induction of oxidative damage, in terms of the production of ROS, in a concentration-dependent manner (Figure 1). A similar observation was recorded after exposure of HepG2 cells to B[a]P [8]. Furthermore, Grauzdytė et al. [18] reported a significant reduction in cell proliferation accompanied by production of oxidative stress in the human bronchial epithelial cells BEAS-2B exposed to PAH extracts. During the metabolism of PAHs, ROS such as superoxide anions, H_2_O_2_, and hydroxyl radicals could be generated [19].

**Figure 2:**
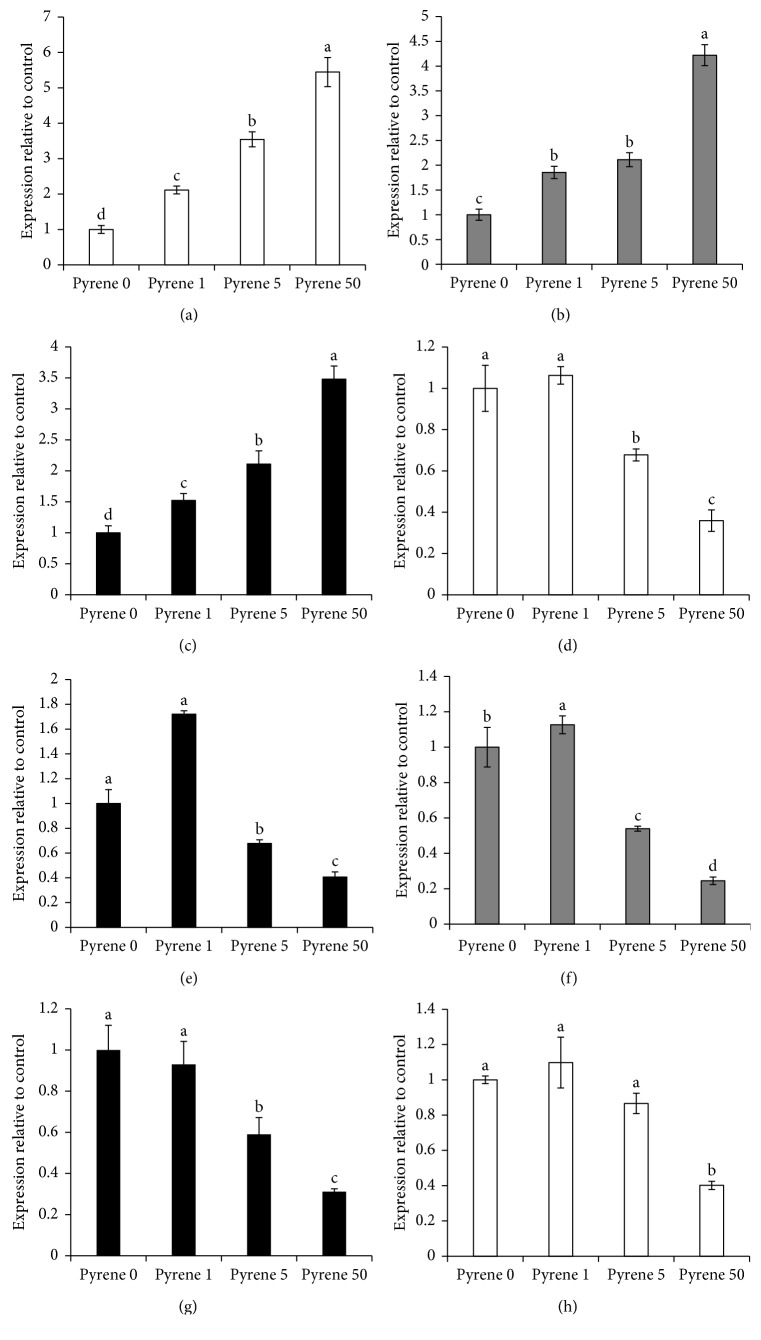
Changes in mRNA expressions of xenobiotic-metabolizing enzymes in HepG2 cells exposed to pyrene. The effects of pyrene (0–50 n) on (a) CYP1A1, (b) CYP1A2, (c) CYP1B1, (d) UGT1A6, (e) GSTA1, (f) NQO1, (g) MDR1, and (h) MRP2 mRNA expression as determined by real-time RT-PCR. Data are presented as mean ± SD (*n* = 6). Columns with different superscript letters are significantly different from each other (*P* < 0.05).

The liver is considered as the major organ for the metabolism and detoxification of xenobiotics including PAHs [20]. Nevertheless, few reports have investigated the effects of pyrene on xenobiotic-metabolizing enzyme systems, and the obtained results indicated a clear induction of phase I enzymes including CYP1A1, 1A2, and 1B1 in a dose-dependent fashion (Figures 2(a)–2(c)). Consistent with this finding, treatment of the human Caco-2 cell line with PAHs, such as B[a]P, chrysene, phenanthrene, benzo[a]fluoranthene, dibenzo[a,b]pyrene, and pyrene, induced mRNA expression of various XMEs, including CYP1A1 and CYP1B1 [21]. Phase II enzymes are mainly involved in the detoxification of the formed metabolites via conjugation reactions [3]. In the present investigation, pyrene significantly downregulated phase II enzymes including UGT1A6, GSTA1, and NQO1 in a dose-dependent manner (Figures 2(d)–2(f)). In agreement with this finding, pyrene modulated the gene expression of UGT1A7 in human Caco-2 cell lines [21]. MDR1 and MRP2 are among the ATP-binding cassette (ABC) transporters that play important roles in the biodetoxification and active efflux of phase II metabolites of drugs and xenobiotics in the body. In the current study, pyrene reduced mRNA expressions of both MDR1 and MRP2 in a dose-dependent manner (Figures 2(g) and 2(h)). In a similar way, either PAH mixture or B[a]P reduced the gene expression of ABC transporters in human colon and liver cells [8, 21].

**Figure 3:**
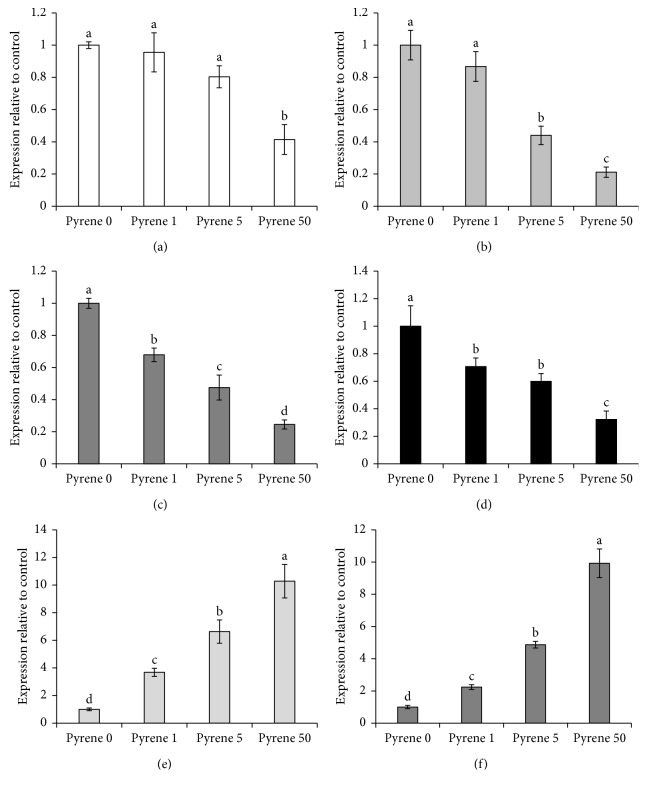
Changes in mRNA expression of antioxidant enzymes and inflammatory markers in HepG2 cells exposed to pyrene. The effects of pyrene (0–50 n) on (a) HO-1, (b) GSTO1, (c) SOD1, (d) CAT, (e) TNF-*α*, and (f) COX2 mRNA expression as determined by real-time RT-PCR. Data are presented as mean ± SD (*n* = 6). Columns with different superscript letters are significantly different from each other (*P* < 0.05).

In order to investigate the possible reasons behind pyrene-induced oxidative damage, the modulatory effects of pyrene on antioxidant enzymes including HO-1, GSTO1, SOD1, and CAT were investigated. Pyrene had clear inhibitory effects on the tested antioxidant enzymes in a dose-dependent manner (Figures 3(a)–3(d)). Similarly, Ajayi et al. [22] reported that B[a]P induced colonic injury via suppression of antioxidant responses in BALB/c mice with clear inhibitory effects on GST- and CAT-dependent enzyme activities. We further investigated the effects of pyrene on the inflammatory response in HepG2 cells; interestingly, inflammatory biomarkers including Cox2 and TNF*α* were significantly induced reaching 10.28 ± 1.21- and 9.92 ± 0.89-fold concentration relative to the control (Figures 3(e) and 3(f)). In agreement with this result, Ferguson et al. [23] reported positive associations between PAHs and plasma inflammation marker C-reactive protein and urinary oxidative stress markers 8-hydroxydeoxyguanosine and 8-isoprostane in pregnant women. In addition, Ajayi et al. [22] confirmed the B[a]P-induced inflammatory response in BALB/c mice. From the overall achieved results in the present study, it is clear that upregulation of phase I enzymes and induction of inflammation together with the downregulation of phase II enzymes and xenobiotic transporters might explain the pyrene-induced cytotoxicity and oxidative stress.

### 3.2. Protective Effects of Lycopene against Pyrene-Induced Adverse Effects in HepG2 Cells

**Figure 4:**
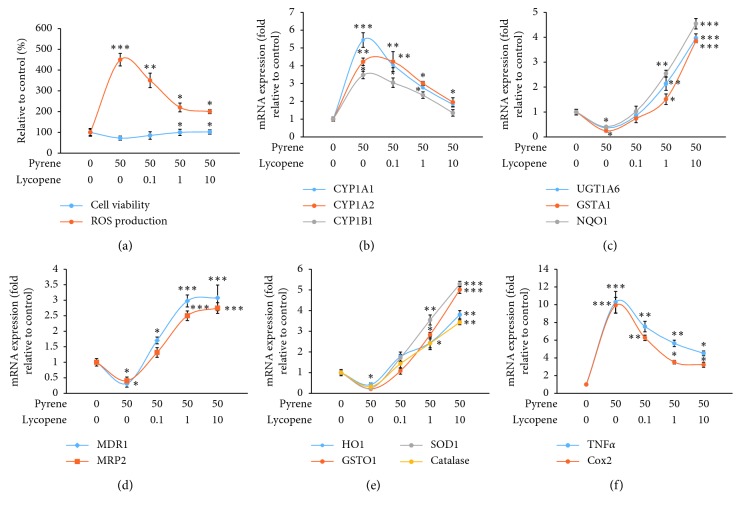
Protective effects of lycopene against pyrene-induced adverse effects in HepG2 cells. Ameliorative effects of lycopene (0–10 *μ*M) on pyrene- (50 n) induced (a) cytotoxicity and oxidative stress, (b) phase I enzymes, (c) phase II enzymes, (d) xenobiotic transporters, (e) antioxidant enzymes, and (f) inflammatory markers. Data are presented as mean ± SD (*n* = 6). Values carrying asterisks (^*∗*^) are different at *P* < 0.05, (^*∗∗*^) are different at *P* < 0.01, and (^*∗∗∗*^) are different at *P* < 0.001, in comparison with the control.

Phytochemicals such as curcumin, resveratrol, quercetin, *β*-carotene, and retinol had been tested for their protective effects against B[a]P-induced genotoxicity and carcinogenicity in lung and liver cells [8, 24]. Lycopene was used as well in combination with either tocopherol or genistein for protection against 7,12-dimethyl[a]benzanthracene-induced oxidative damage and mammary tumorigenesis in female rats [25, 26]. However, the protective effects of lycopene against the adverse effects of pyrene are less informed. In the current investigation, co exposure of HepG2 cells to pyrene and lycopene at three different concentrations showed clear protective effects against pyrene-induced cytotoxicity and oxidative damage (Figure 4(a)). Interestingly, lycopene had clear inhibitory effects against phase I enzymes, including CYP1A1, 1A2, and 1B1 (Figure 4(b)). Coexposure of lycopene with pyrene led to significant induction of phase II enzymes (Figure 4(c)), xenobiotic transporters (Figure 4(d)), and antioxidant enzymes (Figure 4(e)) and subsequently, reduction in inflammatory biomarkers (Figure 4(f)). In this context, carotenoids such as astaxanthin could alter CYP1A dependent activities via induction of protein expression and inhibition of NADPH P450 reductase-dependent electron transfer in male Wistar rats [27]. Furthermore, carotenoids such as *β*-carotene protected HepG2 cells against B[a]P-induced mutagenicity and oxidative stress via upregulation of phase II enzymes and ABC transporters [8]. Similar trends occurred in plants, as application of carotenoids alleviated the oxidative stress caused by phenanthrene in wheat [28]. Epidemiological studies showed a clear inverse relationship between tomato intake and a number of chronic diseases and certain types of cancer, and this was attributed to the higher concentrations of lycopene [29]. Furthermore, supplementation with 2 or 4 mg/kg body weight of lycopene can reduce high-fat diet-induced oxidative stress and liver damage in rats [30]. In addition, Wang et al. reported that lycopene concentrations were elevated in the liver upon repeated exposure for 6 weeks at 15 mg/Kg BW/day from 7.0 ± 1.0 to 17.6 ± 1.5 nmol/g tissue [31]. They added that lycopene supplementation significantly decreased cytochrome P450 2E1, inflammatory foci, and mRNA expression of proinflammatory cytokines (TNF*α*, IL-1, and IL-12), but RF and HO-1 proteins. Therefore, it could be concluded that lycopene-induced upregulation of phase II and III and antioxidant enzymes might be considered as a protection mechanism against pyrene-induced adverse effects.

### 3.3. Effects of Pyrene and Lycopene on Regulatory Elements (Air and RF)

**Figure 5:**
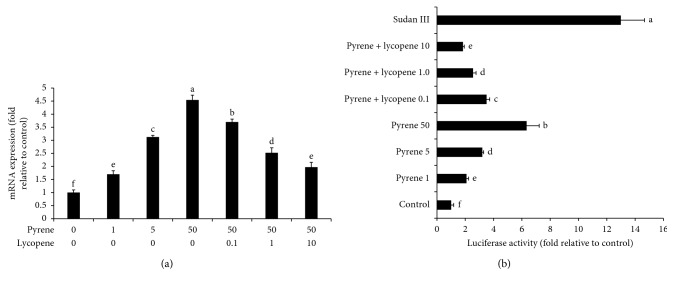
Effects of pyrene and lycopene on Air mRNA expression and luciferase activity. The effects of pyrene and lycopene on (a) Ahr mRNA expression and (b) Ahr luciferase activity. Sudan III was used as a positive control when determining luciferase activity. Data are presented as mean ± SD (*n* = 6). Columns with different superscript letters are significantly different (*P* < 0.05).

PAHs are regulated mainly via Air. This ligand-activated transcription factor regulates the cellular responses to environmental pollutants such as dioxins and PAHs. Air is also involved in the regulation of inflammation as well as a variety of endogenous processes [32]. In order to investigate the possible reasons for upregulation of bioactivating phase I enzymes and inflammatory markers, the effects of pyrene on Ahr mRNA expression and reporter gene activity were studied. Pyrene activated Air at both the transcriptional (∼4.54 ± 0.19 fold) and functional levels (∼6.33 ± 0.88 fold) in human HepG2 cells (Figure 5). Similarly, Air is activated after exposure to PAHs including ketones and quinones [33]. This might explain the upregulation of Air-regulated phase I enzymes including CYP1A1, 1A2, and 1B1 and the inflammatory cytokines including TNF*α* and COX2. In agreement with this assumption, Øvrevik et al. [34] concluded that Air regulates NF-*κ*B signaling and chemokine responses in human bronchial epithelial cells.

**Figure 6:**
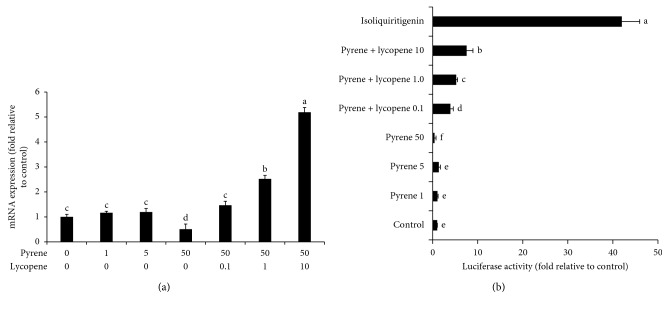
Effects of pyrene and lycopene on RF mRNA expression and luciferase activity. The effects of pyrene and lycopene on (a) RF mRNA expression and (b) RF luciferase activity. Isoliquiritigenin was used as a positive control when determining luciferase activity. Data are presented as mean ± SD (*n* = 6). Columns with different superscript letters are significantly different (*P* < 0.05).

RF is a major transcriptional factor that regulates the cellular response to environmental stressors. It plays an important role in the release of antioxidant detoxification enzymes upon exposure to various xenobiotics. In the current study, the effects of coexposure of lycopene and pyrene on the RF mRNA expression levels and its luciferase activity were investigated. Pyrene alone caused significant reduction in both the expression and activity of RF (Figure 6). Similarly, Wang et al. [35] reported that benzo[a]pyrene reduced mRNA expression of the antioxidant enzymes in the clam *Ruditapes philippinarum* via modulation of the Nrf2-Keap1 signaling pathway. Interestingly, coexposure of pyrene and lycopene could upregulate RF expression and luciferase activity in a dose-dependent manner (Figure 6). These results agree with the results of Abbas et al. [36] who reported that lycopene ameliorates atrazine-induced oxidative damage in the adrenal cortex of male rats by activation of the RF/HO-1 pathway. Furthermore, Yu et al. [37] reported that lycopene attenuates aflatoxin B1-induced renal injury with the activation of the RF antioxidant signaling pathway in mice. Therefore, it is highly suggested that lycopene-induced upregulation of antioxidant enzymes is mechanistically via activation of the RF pathway.

## 4. Conclusion

Pyrene had cytotoxic effects and oxidative damage on human HepG2 cells. This damage is probably due to downregulation of detoxification and antioxidation enzymes. Lycopene could significantly reduce the adverse effects of pyrene on HepG2 cells. Such protective effects of lycopene are possibly via the activation of the RF pathway and reduction of Air metabolic activation of pyrene. Therefore, dietary supplementation of lycopene is highly recommended for people at a high risk for exposure to PAHs such as pyrene.

## Figures and Tables

**Table 1:** Primer sequences of the target genes used in this study.

Target	Primer sequence
CYP1A1	F-5′-CTATCTGGGCTGTGGGCAA-3′
	R-5′-CTGGCTCAAGCACAACTTGG-3′

CYP1A2	F-5′-CATCCC CCACAGCACAACAA-3′
	R-5′-TCCCACTTGGCCAGGACTTC-3′

CYP1B1	F-5′-CTTTCGGCCACTACTCGGAG-3′
	R-5′-CTCGAGGACTTGGCGGCT-3′

UGT1A6	F-5′-CATGATTGTTATTGGCCTGTAC-3′
	R-5′-TCTGTGAAAAGAGCATCAAACT-3′

GSTA1	F-5′-CAGCAAGTGCCAATGGTTGA-3′
	R-5′-TATTTGCTGGCAATGTAGTTGAGAA-3′

NQO1	F-5′-GGATTGGACCGAGCTGGAA-3′
	R-5′-AATTGCAGTGAAGATGAAGGCAAC-3′

MDR1	F-5′-GGGAAGAGCACAACAGTCCA-3′
	R-5′-ATGTGACTGCTGATCACCGC-3′

MRP2	F-5′-AGAGAGCTGCAGAAAGCCAG-3′
	R-5′-CATCTTCCAGGACAAGGGCA-3′

HO1	F-5′-ATGGCCTCCCTGTACCACATC-3′
	R-5′-TGTTGCGCTCAATCTCCTCCT-3′

GSTO1	F-5′-AGGACGCGTCTAGTCCTGAA-3′
	R-5′-TTCCCTGGGTATGCTTCATC-3′

SOD1	F-5′-GCAGGTCCTCACTTTAATCCTCT-3′
	R-5′-ATCGGCCACACCATCTTTGT-3′

CAT	F-5′-TGAAGATGCGGCGAGACTTT-3′
	R-5′-TGGATGTAAAAAGTCCAGGAGGG-3′

TNF*α*	F-5′-GAAGAGTTCCCCAGGGACCT-3′
	R-5′-GGGTTTGCTACAACATGGGC-3′

COX2	F-5′-GAGGGCCAGCTTTCACCAA-3′
	R-5′-TGTGGGAGGATACATCTCTCCA-3′

AhR	F-5ʹ-ATCACCTACGCCAGTCGCAAG-3ʹ
	R-5ʹ-AGGCTAGCCAAACGGTCCAAC-3ʹ

Nrf2	F-5′-CTTGGCCTCAGTGATTCTGAAGTG-3′
	R-5′-CCTGAGATGGTGACAAGGGTTCTA-3′

GAPDH	F-5′-TCCAAAATCAAGTGGGGCGA-3′
	R-5′-TGATGACCCTTTTGGCTCCC-3′

## Data Availability

All analytical data used to support the findings of this study are available from the corresponding author upon request.
